# Supplementary material for: In Vitro Induction of Hypertrophic Chondrocyte Differentiation of Naïve MSCs by Strain
Source: Cells. 2024 Dec 30;14(1):25. doi: 10.3390/cells14010025 (PMC11720650; doi:10.3390/cells14010025)
Supplement: Supplementary file 1 [file cells-14-00025-s001.zip › cells-3365694-supplementary.pdf]

supplementary material

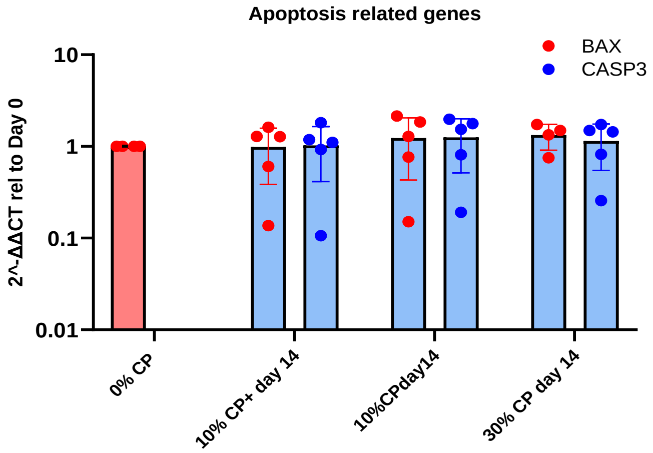

Figure S1: BAX and CASP3 genes expression was quantifies by RT-PCR in samples subjected to 10 or 30% strain in presence of CP or CP+. No significant regulation of these genes were observed between all tested condition and day 0 0% CP control (red bar)

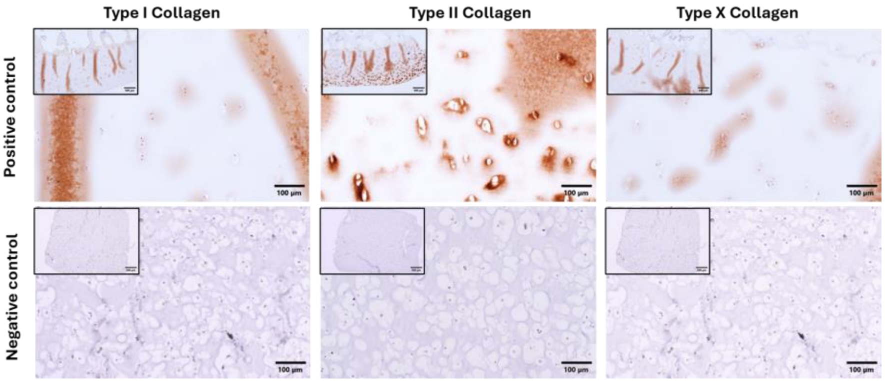

Figure S2: Positive and negative control for Type I, II and X immunostaining. Positive controls were performed on donated native tissue from a 49 years old female patient (cartilage and bone femoral head tissue). Negative control were performed on study samples, omitting the 1st antibody.

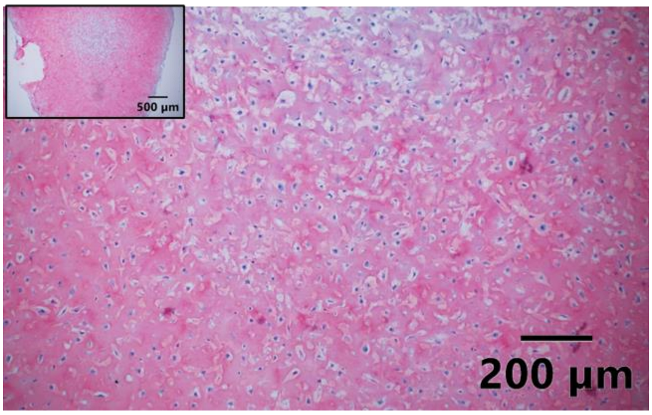

Figure S3: Chondrogenic differentiation potential of MSCs after 14 days static culture in presence of C+ medium (10 ng / mL TGFβ1). Representative picture of 5 tested donors.

Supplemental table S1: Gene array individual genes reference.

Assay ID	Gene Symbol(s)	Gene Name(s)
Hs99999901_s1	18s rRNA	-
Hs00824723_m1	UBC	ubiquitin C
Hs99999902_m1	RPLP0	ribosomal protein lateral stalk subunit P0
Hs00427923_m1	OAZ1	ornithine decarboxylase antizyme 1
Hs00162669_m1	TERT	telomerase reverse transcriptase
Hs04234836_s1	SOX2	SRY-box 2
Hs00171455_m1	LIF	leukemia inhibitory factor
Hs00266645_m1	FGF2	fibroblast growth factor 2
Hs00193291_m1	DLX5	distal-less homeobox 5
Hs00415443_m1	NODAL	nodal growth differentiation factor
Hs00382379_m1	TWIST2	twist family bHLH transcription factor 2
Hs00361186_m1	TWIST1	twist family bHLH transcription factor 1
Hs00234387_m1	CASP3	caspase 3
Hs00171132_m1	GDF15	growth differentiation factor 15
Hs00174128_m1	TNF	tumor necrosis factor
Hs01086867_m1	GDF10	growth differentiation factor 10
Hs00959010_m1	SPP1	secreted phosphoprotein 1
Hs00234160_m1	SPARC	secreted protein acidic and cysteine rich
Hs01045840_m1	VDR	vitamin D (1,25- dihydroxyvitamin D3) receptor
Hs00900055_m1	VEGFA	vascular endothelial growth factor A
Hs01548727_m1	MMP2	matrix metallopeptidase 2
Hs01029144_m1	ALPL	alkaline phosphatase, liver/bone/kidney
Hs00173720_m1	IBSP	integrin binding sialoprotein
Hs00541729_m1	SP7	Sp7 transcription factor
Hs01587814_g1	BGLAP	bone gamma-carboxyglutamate protein
Hs00233476_m1	BMP7	bone morphogenetic protein 7
Hs01028956_m1	COL1A2	collagen type I alpha 2 chain
Hs00231692_m1	RUNX2	runt related transcription factor 2
Hs00610298_m1	FGF10	fibroblast growth factor 10
Hs00410929_m1	SMURF1	SMAD specific E3 ubiquitin protein ligase 1
Hs00224203_m1	SMURF2	SMAD specific E3 ubiquitin protein ligase 2
Hs01016882_m1	CALCR	calcitonin receptor
Hs00228830_m1	SOST	sclerostin
Hs00154192_m1	BMP2	bone morphogenetic protein 2
Hs00179899_m1	MGP	matrix Gla protein
Hs00915142_m1	FGFR1	fibroblast growth factor receptor 1
Hs00831730_s1	BMPR1A	bone morphogenetic protein receptor type 1A
Hs00932129_m1	COL9A1	collagen type IX alpha 1
Hs01552926_m1	FGFR2	fibroblast growth factor receptor 2
Hs00179829_m1	FGFR3	fibroblast growth factor receptor 3
Hs00234994_m1	PDGFA	platelet derived growth factor subunit A
Hs01029057_m1	MMP8	matrix metallopeptidase 8
Hs00176676_m1	KDR	kinase insert domain receptor
Hs01099594_m1	BMP6	bone morphogenetic protein 6
Hs00181829_m1	FGF9	fibroblast growth factor 9
Hs00609088_m1	COL5A1	collagen type V alpha 1
Hs00195437_m1	SMAD5	SMAD family member 5
Hs00969210_m1	SMAD3	SMAD family member 3
Hs00929647_m1	SMAD4	SMAD family member 4
Hs00998188_mH	SMAD2	SMAD family member 2
Hs00195432_m1	SMAD1	SMAD family member 1
Hs00959143_m1	BGN	biglycan
Hs01008571_m1	LCN2	lipocalin 2
Hs00374709_m1	SOX5	SRY-box 5
Hs00264525_m1	SOX6	SRY-box 6
Hs00165814_m1	SOX9	SRY-box 9
Hs00153936_m1	ACAN	aggrecan
Hs00264051_m1	COL2A1	collagen type II alpha 1 chain
Hs00942584_m1	MMP13	matrix metallopeptidase 13
Hs00370078_m1	BMP4	bone morphogenetic protein 4
Hs00998133_m1	TGFB1	transforming growth factor beta 1
Hs00234244_m1	TGFB2	transforming growth factor beta 2
Hs01086000_m1	TGFB3	transforming growth factor beta 3
Hs00610320_m1	TGFBR1	transforming growth factor beta receptor 1
Hs00234253_m1	TGFBR2	transforming growth factor beta receptor 2
Hs00176148_m1	BMPR2	bone morphogenetic protein receptor type 2
Hs03044164_m1	BAMBI	BMP and activin membrane bound inhibitor
Hs03676575_s1	ID1	inhibitor of DNA binding 1, HLH protein
Hs00968305_m1	MMP3	matrix metallopeptidase 3
Hs00153181_m1	EGF	epidermal growth factor
Hs00981633_m1	PRG4	proteoglycan 4
Hs01091999_m1	HAPLN1	hyaluronan and proteoglycan link protein 1
Hs00174131_m1	IL6	interleukin 6
Hs00961622_m1	IL10	interleukin 10
Hs00167060_m1	GDF5	growth differentiation factor 5
Hs00953798_m1	ACVRL1	activin A receptor like type 1
Hs01095585_m1	COL6A1	collagen type VI alpha 1
Hs00166657_m1	COL10A1	collagen type X alpha 1 chain
Hs01081801_m1	IHH	indian hedgehog
Hs00246364_m1	DMRT2	doublesex and mab-3 related transcription factor 2
Hs00174969_m1	PTHLH	parathyroid hormone like hormone
Hs00164359_m1	COMP	cartilage oligomeric matrix protein
Hs00153133_m1	PTGS2	prostaglandin-endoperoxide synthase 2
Hs00998193_m1	SMAD7	SMAD family member 7
Hs01556515_m1	TCF7	transcription factor 7 (T-cell specific, HMG-box)

A Applied Biosystem:

Gene Name	Assay ID
OAZ1	Hs00427923_m1
ALP	Hs00758162_m1
Type II Collagen (alpha1)	Hs00264051_m1
COMP	Hs00164359_m1
SOX9	Hs00165814_m1

B Microsynth:

Gene Name	Forward	Reverse	Probe
RPLP0	5'-TGG GCA AGA ACA CCA TGA TG-3'	5'-CGG ATA TGA GGC AGC AGT TTC-3'	5'-AGG GCA CCT GGA AAA CAA CCC AGC-3'
Type I Collagen (alpha1)	5'-CCC TGG AAA GAA TGG AGA TGA T-3'	5'-ACT GAA ACC TCT GTG TCC CTT CA-3'	5'-CGG GCA ATC CTC GAG CAC CCT -3'
Type X Collagen (alpha1)	5'-ACG CTG AAC GAT ACC AAA TG-3'	5'-TGC TAT ACC TTT ACT CTT TAT GGT GTA-3'	5'-ACT ACC CAA CAC CAA GAC ACA GTT CTT CAT TCC-3'
ACAN	5'-AGT CCT CAA GCC TCC TGT ACT CA-3'	5'-CGG GAA GTG GCG GTA ACA-3'	5'-CCG GAA TGG AAA CGT GAA TCA GAA TCA ACT-3'
MMP13	5'-CGG CCA CTC CTT AGG TCT TG-3'	5'-TTT TGC CGG TGT AGG TGT AGA TAG-3'	5'-CTC CAA GGA CCC TGG AGC ACT CAT GT-3'
RUNX2	5'-AGC AAG GTT CAA CGA TCT GAG AT-3'	5'-TTT GTG AAG ACG GTT ATG GTC AA-3'	5'-TGA AAC TCT TGC CTC GTC CAC TCC G-3'
VEGFA	5'-GCC CAC TGA GGA GTC CAA CA-3'	5'-TCCTATGTG CTG GCC TTG GT-3'	5'-CAC CAT GCA GAT TAT GCG GAT CAA ACC T-3'

Supplemental table S2 A and B: (A) Applied Biosystem gene expression assays, (B) Forward, Reverse and Prode sequences of Microsynth genes assays.

	2^-ΔΔCT **10% CP day14**	2^-ΔΔCT **30% CP day14**	2^-ΔΔCT **10% CP+ day14**
**PIEZO2#**	* p=0.0179	ns p=0.4643	* p=0.0179
**TRPV4$**	* p=0.0286	* p=0.0286	* p=0.0143
**TAZ$**	* p=0.0163	* p=0.0403	ns p=0.2108
**YAP#**	ns p=0.3413	ns p=0.4643	ns p=0.3328
**PIEZO1#**	ns p=0.5105	ns p=0.2625	ns p=0.3328
**RSPO1#**	** p=0.0079	ns p=0.0635	ns p=0.0635
**WNT8A#**	* p=0.0143	ns p=0.1657	* p=0.0143
**WNT4#**	ns p=0.4643	ns p=0.0635	* p=0.0476
**WNT5A#**	ns p=0.0671	ns p=0.0898	* p=0.0123
**LRP5 #**	** p=0.004	ns p=0.0635	ns p=0.0635

Supplemental table S3: p values of heatmap data reported in Figure 3. According to normal distribution of the data, two-way ANOVA with multiple comparison ($) or Kruskal-Wallis with Dunn's correction (#) was used.
